# *RPL13A* and *EEF1A1* Are Suitable Reference Genes for qPCR during Adipocyte Differentiation of Vascular Stromal Cells from Patients with Different BMI and HOMA-IR

**DOI:** 10.1371/journal.pone.0157002

**Published:** 2016-06-15

**Authors:** Adriana-Mariel Gentile, Said Lhamyani, Leticia Coín-Aragüez, Wilfredo Oliva-Olivera, Hatem Zayed, Antonio Vega-Rioja, Javier Monteseirin, Silvana-Yanina Romero-Zerbo, Francisco-José Tinahones, Francisco-Javier Bermúdez-Silva, Rajaa El Bekay

**Affiliations:** 1 IBIMA, Universidad de Málaga, Campus Teatinos s/n, 29010, Málaga, España; 2 CIBER Pathophysiology of obesity and nutrition CB06/03, Carlos III Health Institute, Malaga, 29010, Spain, Laboratory of Biomedical Research, Virgen de la Victoria Clinical University Hospital, Málaga, 29010, Spain; 3 Biomedical Sciences Program, Health Sciences Department, College of Arts and Sciences, Qatar University, P.O. Box 2713, Doha, Qatar; 4 Unidad de Gestión Clínica de Alergia Intercentros, Hospital Universitario Virgen Macarena, Sevilla, Spain; 5 Unidad de Gestion Clínica Intercentros de Endocrinología y Nutrición, Instituto de Investigación Biomédica de Málaga (IBIMA), Hospital Regional Universitario de Málaga/Universidad de Málaga, 29009, Málaga, Spain; 6 Centro de Investigación Biomédica en Red de Diabetes y Enfermedades Metabólicas Asociadas (CIBERDEM), Málaga, Spain; 7 Endocrinology Service, Virgen de la Victoria Clinical University Hospital, Malaga, 29010, Spain; Naval Research Laboratory, UNITED STATES

## Abstract

Real-time or quantitative PCR (qPCR) is a useful technique that requires reliable reference genes for data normalization in gene expression analysis. Adipogenesis is among the biological processes suitable for this technique. The selection of adequate reference genes is essential for qPCR gene expression analysis of human Vascular Stromal Cells (hVSCs) during their differentiation into adipocytes. To the best of our knowledge, there are no studies validating reference genes for the analyses of visceral and subcutaneous adipose tissue hVSCs from subjects with different Body Mass Index (BMI) and Homeostatic Model Assessment of Insulin Resistance (HOMA-IR) index. The present study was undertaken to analyze this question. We first analyzed the stability of expression of five potential reference genes: *CYC*, *GAPDH*, *RPL13A*, *EEF1A1*, and *18S* ribosomal RNA, during *in vitro* adipogenic differentiation, in samples from these types of patients. The expression of *RPL13A* and *EEF1A1* was not affected by differentiation, thus being these genes the most stable candidates, while *CYC*, *GAPDH*, and *18S* were not suitable for this sort of analysis. This work highlights that *RPL13A* and *EEF1A1* are good candidates as reference genes for qPCR analysis of hVSCs differentiation into adipocytes from subjects with different BMI and HOMA-IR.

## Introduction

The mechanisms underlying insulin resistance (IR) and type 2 diabetes (T2D) development in obese subjects are not fully understood. Thus, there is currently an intense research aiming to understand the mechanisms underlying these processes [1,2]. Several lines of evidence indicate that the physiopathology of these diseases involves mechanisms other than only fat accumulation. Indeed, this paradox can be seen in the fact that some morbidly obese subjects do not develop IR and/or diabetes, whereas some lean subjects do. In this line, several studies have been focused on the analysis of adipose tissue (AT) expansion capacity as one of the driving factors in the development of these metabolic disorders. The expandability hypothesis states that the capacity to expand of the fat depots are not the same for all subjects and once the limit of storage capacity is exceeded, the lipids are accumulated ectopically in other organs, inducing the secretion of pro-inflammatory factors that drive to insulin resistance and further T2D development [3,4]. This expansion capacity depends on the resident stem cells and on their capacity to differentiate into adipocyte cell lineages [3,5]. Gene expression analyses by qPCR are among the current widely used techniques to investigate these mechanisms, being necessary to reliably detect small changes in gene expression during the AT expansion and adipocyte differentiation. Reference genes are commonly used as internal controls to quantify changes in mRNA levels [6]. These controls are required to avoid variations in RNA quality, content and stability, reaction efficiency, and sample loading [7]. This variability can also be further increased among samples from different subjects, tissues and time courses, leading to data misinterpretation. Therefore, it is peremptory to identify and validate reliable reference genes for qPCR analysis [8]. Given that there is no universal reference gene for all biological processes [9], the use of multiple stable reference genes is the widely accepted method for qPCR data normalization [8,10,11].

*GAPDH* and *β-Actin* are the most widely used reference genes. However, their expression is known to be influenced by developmental and environmental factors. Other studies have used *GAPDH* and Ribosomal Protein L13A (*RPL13A*) for adipogenesis and osteogenesis in bone marrow stem cell differentiation [12,13]. Other study has used *RPL13A* and *EF1α* for Marrow-Isolated Adult Multilineage Inducible (MIAMI) and Recycling Stem (RS-1) cells [14]. *RPL13A* was also shown to be reliable for the analysis of bone marrow- and placenta-derived MSCs during expansion, adipo-, chondro-, and osteo-genesis, in primary Human Bone Cells (HBCs), and in the osteosarcoma cell line MG-63 [15]. Amable *et al*. studied five genes in human MSCs from liposuction of abdominal fat, and found that *RPL13A* was the most appropriate reference gene [11]. The *18S* gene has been used as reference for human subcutaneous adipose tissue derived pre-adipocytes [16,17]. However, its expression stability has not been yet validated. Ferguson *et al*. studied the expression of six genes in the 3T3-L1 cell line under four experimental conditions (inflammatory stress, oxidative stress, cell cycle progression and differentiation), and found that the *18S* is the most reliable reference gene [18].

To date, there are no validated reference genes for adipogenic differentiation of mesenchymal cells either from human Visceral Adipose Tissue (VAT) and Subcutaneous Adipose Tissue (SAT) isolated from subjects with different BMI and HOMA-IR. Given the importance of the understanding of the mechanisms involved in the adipogenic differentiation in human AT, and the potential variability of reference genes depending on the source of this tissue and disease status, it is essential to have available validated reference genes to reliably study these processes. The present work was undertaken to analyze this question.

## Materials and Methods

### Patients

AT samples were extracted from morbidly obese patients (n = 8) undergoing bariatric surgery at the Virgen de la Victoria Clinical University Hospital (Malaga, Spain). Exclusion criteria: Subjects with diabetes mellitus type 2 treated with insulin, with cardiovascular disease in the 6 months prior to the inclusion in the study, with any evidence of acute or chronic inflammatory disease, with infectious disease or patients’ refusal to participate in the study. Non-morbidly obese subjects (n = 8) who underwent laparoscopic surgery for hiatus hernia or cholelithiasis, matched by age to the obese group, acted as controls. Exclusion criteria for control subjects were the same as for the morbidly obese patients. The study groups were classified as follows: Lean/L-IR; subjects with BMI 18.5–24.9 and a low degree of insulin resistance (HOMA-IR<3.5), Lean/H-IR; subjects with BMI 18.5–24.9 and a high degree of insulin resistance (HOMA-IR>7), MO/L-IR; morbidly obese subjects (BMI>40) with a low degree of insulin resistance (HOMA-IR<3.5), MO/H-IR; morbidly obese subjects (BMI>40) with a high degree of insulin resistance (HOMA-IR>7). The cut-off point for HOMA-IR was set at 3.5 using 90^th^ percentile criteria in a Spanish population [19]. All participants gave their written informed consent, and the study was reviewed and approved by the Ethics and Research Committee of the Virgen de la Victoria Clinical University Hospital. The average age of each group and their anthropometric and biochemical characteristics are shown in Table 1. Both subcutaneous and visceral adipose tissues (SAT and VAT) were obtained at the beginning of the surgical procedure and were stored immediately at -80°C.

**Table 1:** Anthropometric, clinical and metabolic characterization of patients. Donors (n = 16) were selected according to BMI and HOMA-IR. Data are expressed as the mean ± SEM. Comparison among groups was performed by Kruskal-Wallis and Mann-Whitney tests. Note that in each arrow, "a" and "b" letters in superscript represent statistically different groups. L-IR: Low-degree insulin resistance, H-IR: High-degree insulin resistance. DAP: Diastolic Arterial Pressure, SAP: Systolic Arterial pressure.

	Lean L-IR (n = 4)	Lean H-IR (n = 4)	Morbid obese MO- L-IR (n = 4)	Morbid obese MO- H- IR (n = 4)
**Age (Years)**	51.68±15.96	47.53±12.11	43.64±10.70	38.42±9.20
**BMI**	23.11±1.59^a^	24.21±1.13^a^	57.13±8.40^b^	57.59±6.60^b^
**Waist Circumference(cm)**	83.76±7.90^a^	85.12±5.40^a^	142.80±21.77^b^	149.00±25.53^b^
**Triglycerides (mM)**	1.16±0.53^a^	1.64±1.21^b^	1.19±0.50^a^	1.67±1.05^b^
**Cholesterol (mM)**	5.20±1.18	4.77±1.01	5.39±1.52	4.96±0.67
**HDL cholesterol (mM)**	1.47±0.52^a^	1.52±0.41^a^	0.93±0.68^b^	0.98±0.47^b^
**SAP (mmHg)**	121.47±11.78	129.67±10.71	134.40±22.51	142.67±25.87
**DAP(mmHg)**	68.47±9.91^a^	72.23±8.18^a^	82.40±10.90^b^	86.33±17.28^b^
**HOMA IR**	1.11±0.84^a^	10.33±4.52^b^	3.32±0.82^a^	12.54±4.97^b^
**Adiponectin (μg/mL)**	13.55±5.81^a^	14.65±4.76^a^	10.83±4.88^b^	9.21±4.90^b^

### AT collection and experimental design

The stromal vascular fraction (SVF) was isolated from VAT (n = 16) and SAT (n = 16) immediately after their extraction. SVF cells were *in vitro* induced to differentiate into adipocytes and the total mRNA was isolated from the 64 samples (32 from undifferentiated cells and 32 from differentiated adipocytes). Adipocyte differentiation was assessed by oil red O-staining, which was performed by fixing the cells in neutral buffered formalin, followed by staining of intracellular lipid droplets with a 30% in PBS solution of Oil Red O (Thermo Fisher (Kandel) GmbH, Karlsruhe, Germany), prepared from a stock solution of Oil Red O 0.5% in isopropanol, and by the mRNA expression of the adipocyte markers *FABP4* and *PPARγ2*. Ct analysis for *CYC*, *GAPDH*, *RPL13A*, *EEF1A1*, and *18S* genes was performed together with the analysis of the change in Ct during differentiation. By this way, analysis of gene expression stability among the studied groups and during differentiation was assessed. Finally, validation of our findings was performed by the Bestkeeper algorithm and RefFinder platform.

### Isolation of SVF from VAT and SAT

The reagents were purchased from Sigma (St. Louis, MO, USA) unless otherwise stated. The Isolation of SVF from VAT and SAT was carried out using a modified procedure from Zuk *et al*. [20]. Briefly, AT samples were transported in Hank's balanced salt solution supplemented with penicillin and streptomycin. Samples were washed twice with PBS, fragmented, and enzymatically digested in a solution containing type II collagenase and bovine serum albumin (BSA) for 20 min at 37°C on a shaking water bath. The resulting cell suspension was centrifuged at 500*xg* for 10 min. Floating adipocytes were discarded and the precipitate containing the SVF was filtered through a 100 μm mesh, and centrifuged at 400*xg* for 5 min. The cell pellets were resuspended in an hypotonic buffer to lysate the erythrocytes for 10 min at room temperature and centrifuged again at 400*xg* for 5 min. Cell pellets were then suspended in expansion medium DMEM/F12 supplemented with 10% fetal bovine serum, 100 μg/ml streptomycin, 100 U/ml penicillin, 2 mM L-glutamine, and 1 μg/ml amphotericin B. Cells were then plated into tissue culture flasks and incubated at 37°C in a humid atmosphere with 5% of CO_2_ for approximately 8 days until 90% of confluence was reached.

### Adipogenic differentiation

Adipogenic differentiation was carried out as previously described [21,22]. Briefly, cells were induced to differentiate between passages two and three. SVF cells from both tissues were seeded in 12 and 96 well plates at a density of 10.000 cells per cm^2^ in human preadipocyte medium (DMEM/Hams F-12 medium (1:1, vol/vol), 20mM HEPES pH 7.4 supplemented with 10% fetal bovine serum, 1% penicillin and streptomycin). The preadipocyte medium was replaced every 2–3 days upon reaching 90% confluence and then replaced with adipogenic medium on the 21^st^ day. During the first three days the induction medium was DMEM/Hams F-12 medium (1:1, vol/vol), 20 mM HEPES pH 7.4 supplemented with 10% fetal bovine serum, 33μM biotin, 17μM pantothenate, 10μM human insulin, 1μM dexamethasone, 0.5mM IBMX, 10μM pioglitazone, 0.5μM rosiglitazone, 1% penicillin/streptomycin and 1μg/ml amphotericin B. Then, the medium was replaced by adipogenic medium: DMEM/Hams F-12 medium (1:1, vol/vol), 20mM HEPES pH 7.4 supplemented with 10% fetal bovine serum, 10μM human insulin, 1μM dexamethasone, 10μM pioglitazone, 0.5μM rosiglitazone. After one week of the adipocyte differentiation, cells were collected and processed for mRNA extraction and qPCR.

### mRNA extraction and qPCR

Total RNA was isolated using the RNA Stat 60 Reagent (Ams Biotechnology, Abingdon, UK). Total RNA was quantified using NanoDrop ND-1000 spectrophotometer V3.7.1 (Thermo Scientific, Massachusetts, MA, USA). RNA purity was assessed by checking the absorbance at 260 nm, 280 nm and 230 nm. A ratio A260/A280<1.8 indicates protein contamination and <2 presence of phenol, chaotropic salts (guanidinium thiocyanate) or proteins.

RNA was reverse transcribed using Transcriptor reverse Transcriptase (Roche Diagnostic, Barcelona, Spain). cDNA amplifications were carried out using a MicroAmp^®^ fast optical 96-well reaction plate (Applied Biosystems, CA, USA) on an Applied Biosystems 7500 Fast Real-Time PCR System. Quantitative RT-PCR reactions were carried out for all genes using specific TaqMan^®^ probes (Applied Biosystems, CA, USA) (S1 Table). PCR reactions were run in duplicate. A PCR negative control (without template) and positive control (with a template of known amplification) were included in each assay. During the PCR, the Ct values for each amplified product were determined using a threshold value of 0.1.

### Determination of the expression and stability of the reference genes

To determine the expression and stability of the candidate reference genes in the different groups and during differentiation, two different approaches were used: Firstly, we performed a Ct analysis over the raw non-normalized data and assessed the changes in ΔCt during adipogenesis and, secondly, we validated the findings by the Bestkeeper algorithm and RefFinder platform. For the study of putative changes in reference genes during differentiation, they were analyzed and validated using the criterion of ΔCt value ≤ ±0.5 as a delimiter of reference gene suitability. For each tissue sample, expression stability of each gene was calculated using the mean of the Ct values and all validation data were converted into fold-changes using the formula 2^-ΔCt^. It is widely accepted that reference gene expression levels that fall in between 0.7 and 1.4 are considered fluctuation in gene expression that are due to technical variance [23]. BestKeeper is an excel-based tool using pair-wise correlations which determines the best suited standards, out of ten candidates, and combines them into an index, being one of the most appropriate tool for the validation of a first screening [24]. The Bestkeeper software was downloaded from http://www.gene-quantification.de/bestkeeper.html and used according to the developer’s instructions. BestKeeper identifies the reference genes when they exhibit the lowest Standard Deviation (SD) and highest Pearson correlation coefficient (r). Genes that show a SD greater than 1 are considered unacceptable [24]. The BestKeeper index is the geometric mean of the Ct values of the highly correlated candidate reference gene. Descriptive statistics of the derived crossing points are calculated for each reference gene: the Geometric Mean (GM), Arithmetic Mean (AM), Minimal (Min) and Maximal (Max) value, Standard Deviation (SD), and Coefficient of Variance (CV). All crossing point data are compared over the entire study, considering the different groups. The X-fold over- or under-expression of individual samples towards the geometric mean crossing point (2^-ΔCt^) are calculated, and the multiple factors of their minimal and maximal values are expressed as the x-fold ratio and its standard deviation. RefFinder, which integrates the currently available major computational softwares (geNorm, Normfinder, BestKeeper, and the comparative Ct method), was used to verify the comparative results obtained from Bestkeeper and ΔCt analysis carried out for this work [25]. RefFinder was downloaded from http://fulxie.0fees.us/ and used according to the developer’s instructions.

### Statistical analysis

**Fig 1:**
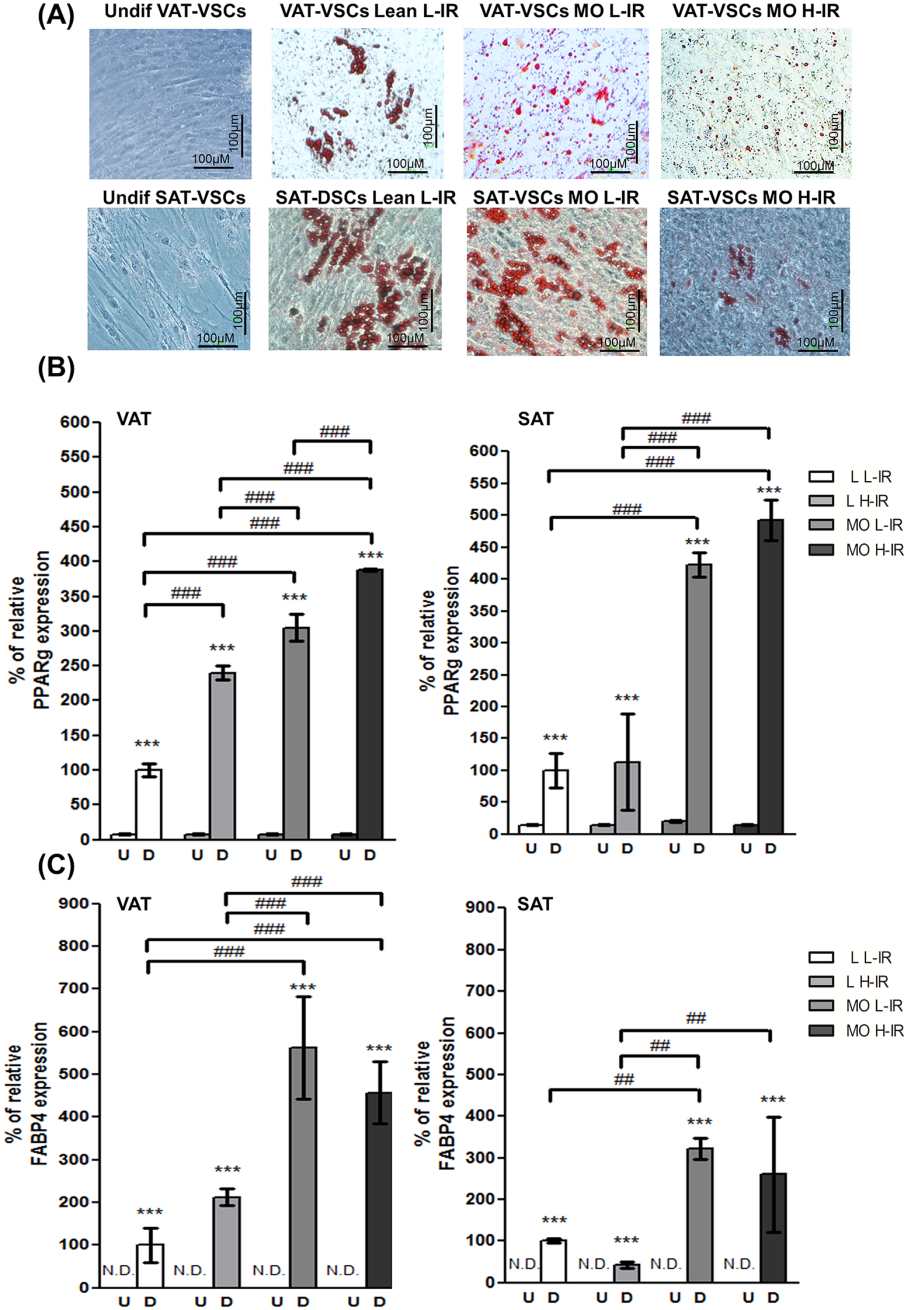
Adipogenic differentiation of hVSCs from VAT and SAT of subjects with different BMI and HOMA-IR index. A) Representative photomicrographs of undifferentiated VAT-derived stromal cells (VAT-VSCs) and SAT-derived stromal cells (SAT-VSCs) (left column) and differentiated adipocytes from VAT-VSCs (upper row) and SAT-VSCs (lower row) stained with the lipophilic marker oil red O. The images show brown spots in samples from all groups, corresponding to lipid accumulation within the newly generated adipocytes. Due to a technical problem, images from Lean H-IR samples are not available. They were instead analyzed by RT-PCR. (B and C) mRNA expression of the adipocyte marker *PPARγ2* (B) and *FABP4* (C) in undifferentiated and differentiated samples corresponding to VAT (left column) and SAT (right column) from all groups. Gene expression was referred to differentiated lean-Low-IR samples, which were considered as 100%; *RPL13A* was used as reference gene. Data represent mean ± SEM of four samples in each bar. *FABP4* expression in undifferentiated samples could not be detected in our assay (N.D.). Both *PPARγ2* and *FABP4* expression were dramatically increased in samples from differentiated cells, being higher in samples from morbid obese when compared to lean subjects, independently of the associated insulin resistance condition, (MO L-IR or MO H-IR versus Lean L-IR or Lean H-IR). Additionally, *PPARγ2* levels in VAT were different in each group when compared to any other, with expression levels decreasing in the following way, MO H-IR>MO L-IR>Lean H-IR>Lean L-IR (Two-way ANOVA and Bonferroni post-hoc test. Asterisks represent comparisons between undifferentiated and differentiated samples within each study group; hashes represent comparisons among differentiated samples from different study groups). VAT: visceral adipose tissue; SAT: subcutaneous adipose tissue; VAT-VSCs: stromal cells derived from visceral adipose tissue; SAT-VSCs: stromal cells derived from subcutaneous adipose tissue; Lean L-IR: lean subjects with low-degree insulin resistance; Lean H-IR: lean subjects with high-degree insulin resistance; MO L-IR: morbid obese subjects with low-degree insulin resistance; MO H-IR: morbid obese with high-degree insulin resistance.

**Fig 2:**
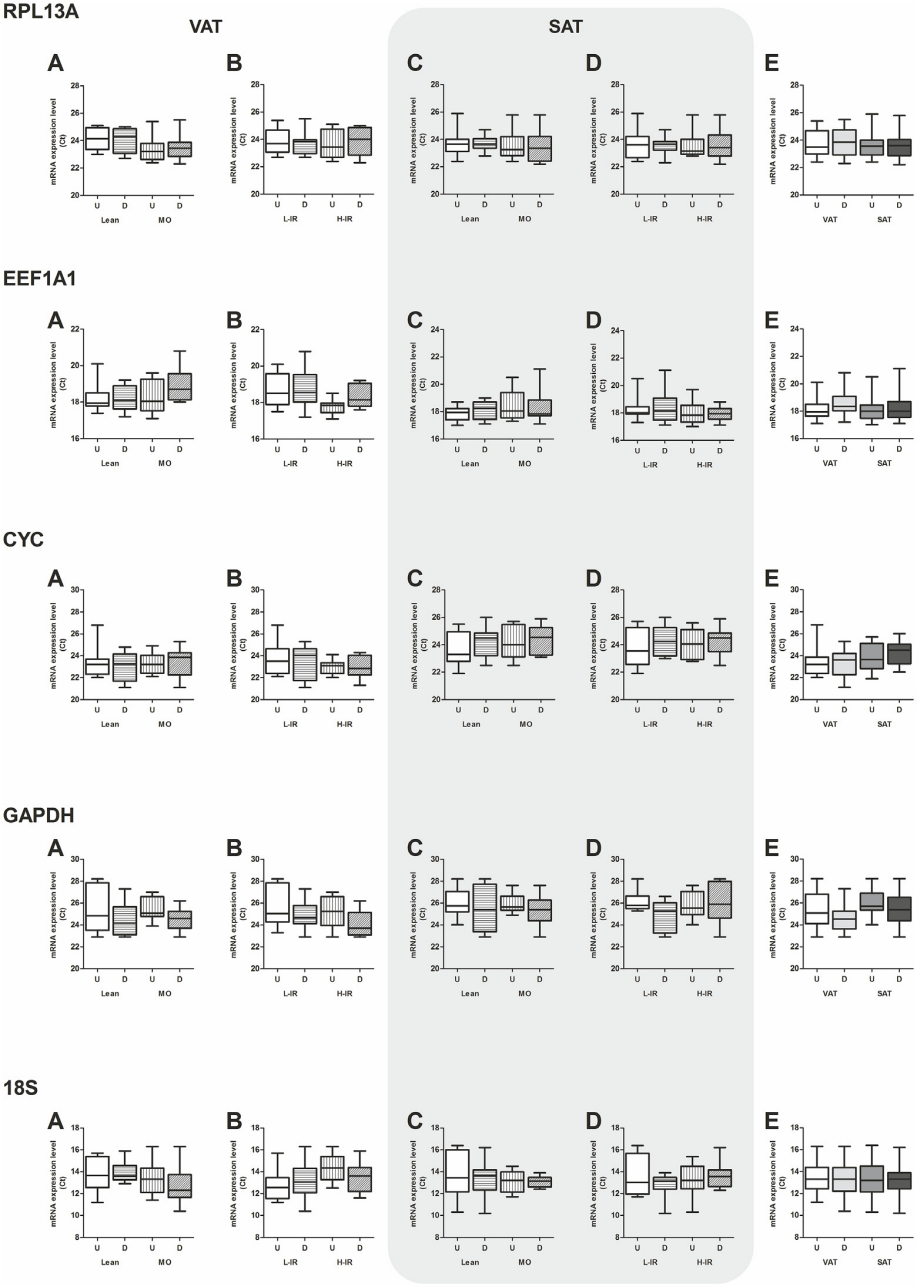
Boxplots of mRNA expression for *RPL13A*, *EEF1A1*, *CYC*, *GAPDH* and *18S* from undifferentiated and differentiated cells. Raw non-normalized Ct values from qPCR were collected and analyzed to look for differences in absolute gene expression among groups and fat depots. Data were grouped to compare Ct of lean versus morbid obese (low-IR + high-IR) in VAT (A) and SAT (C), low-IR versus high-IR (lean + morbid obese) in VAT (B) and SAT (D), and VAT versus SAT (E). Ct values are represented in boxplots as median and 2.5–97.5 percentile; n = 8 samples in each group except in VAT versus SAT, where n = 16 in each group. No changes among groups were detected for any tissue and reference gene investigated. Two-way ANOVA. VAT: visceral adipose tissue; SAT: subcutaneous adipose tissue. MO: morbid obese; L-IR: low-degree insulin resistance; H-IR: high-degree insulin resistance.

Clinical parameters and gene expression data are expressed as means ± SEM, except Ct data which have been represented by boxplots with median and 97.5 percentile. Normality tests were performed by Shapiro-Wilk test. The Levene’s test was used to determine homogeneity of variance. Kruskal-Wallis and Mann-Whitney tests were used to compare mean among the groups presented in Table 1, and one-way or two-way ANOVA test, following Bonferroni post-hoc test to the data of Table 2 and Figs 1 and 2. In all cases, the rejection levels for a null hypothesis were α = 0.05 for two tails. All statistical analysis were performed using the IBM SPSS Statistic software program SPSS (v22.0. for Windows; Chicago, IL, USA).

**Table 2:** Descriptive analysis of Ct values variability from qPCR of Undifferentiated and differentiated hVSCs from VAT of patients with different BMI and HOMA-IR index. The table shows the statistics min, max, mean ± SEM, standard variation (SD) and variance of the Ct values obtained after qPCR of undifferentiated and differentiated VAT-derived hVSCs in the four groups under investigation as well as the mean values for every reference gene assessed (right columns). Data show that *RPL13A* and *EEF1A1* has the lowest variability (SD and variance values) suggesting more stability in their expression when compared to the other reference genes. VAT: visceral adipose tissue; SAT: subcutaneous adipose tissue. Lean L-IR: lean subjects with low-degree insulin resistance; Lean H-IR: lean subjects with high-degree insulin resistance; MO L-IR: morbid obese subjects with low-degree insulin resistance; MO H-IR: morbid obese with high-degree insulin resistance.

RPL13A in VAT									
	Lean L-IR		Lean H-IR		MO L-IR		MO H-IR		MEAN
	Undiff	Diff	Undiff	Diff	Undiff	Diff	Undiff	Diff	
Min	22.99	22.68	23.49	24.59	22.66	23.49	22.36	22.3	23.07
Max	24.82	24.01	25.14	24.95	25.41	25.48	23.42	23.38	24.58
Mean ±SEM	23.85±0.43	23.35±0.35	24.4±0.4	24.8±0.08	23.88±0.57	24.16±0.45	22.84±0.24	22.91±0.26	23.77±0.35
SD	0.85	0.71	0.81	0.16	1.15	0.89	0.47	0.52	**0.70**
Variance	0.73	0.5	0.66	0.027	1.31	0.8	0.22	0.27	**0.56**
EEF1A1 in VAT									
	Lean L-IR		Lean H-IR		MO L-IR		MO H-IR		MEAN
	Undiff	Diff	Undiff	Diff	Undiff	Diff	Undiff	Diff	
Min	17.79	17.19	17.38	17.58	17.53	18.03	17.05	18.10	17.58
Max	20.13	19.02	18.02	19.22	19.58	20.78	18.51	19.05	19.29
Mean ±SEM	18.73±0.49	18.23±0.39	17.77±0.14	18.16±0.37	18.69±0.5	19.23±0.62	17.75±0.31	18.57±0.24	18.39±0.38
SD	0.99	0.78	0.28	0.74	0.99	1.24	0.61	0.49	**0.77**
Variance	0.98	0.62	0.08	0.55	0.98	1.53	0.37	0.24	**0.67**
CYC in VAT									
	Lean L-IR		Lean H-IR		MO L-IR		MO H-IR		MEAN
	Undiff	Diff	Undiff	Diff	Undiff	Diff	Undiff	Diff	
Min	22.13	21.12	22.04	21.34	22.12	21.12	22.16	22.07	21.76
Max	26.76	24.84	23.35	23.63	24.86	25.28	24.07	24.29	24.64
Mean ±SEM	23.98±0.99	23.53±00.82	22.87±0.29	22.65±0.48	23.52±0.58	23.53±0.88	23.1±0.39	23.32±0.55	23.31±0.62
SD	1.98	1.65	0.59	0.95	1.15	1.75	0.78	1.11	**1.25**
Variance	3.92	2.71	0.34	0.91	1.33	3.07	0.62	1.23	**1.77**
GAPDH in VAT									
	Lean L-IR		Lean H-IR		MO L-IR		MO H-IR		MEAN
	Undiff	Diff	Undiff	Diff	Undiff	Diff	Undiff	Diff	
Min	23.26	24.61	22.87	22.91	24.79	22.88	23.86	23.59	23.60
Max	28.23	27.30	26.84	23.71	26.81	25.12	26.95	26.23	26.40
Mean ±SEM	25.95±1.32	25.63±0.64	24.85±0.86	23.30±0.22	25.43±0.47	24.11±0.47	25.41±0.67	24.93±0.55	24.95±0.65
SD	2.63	1.28	1.73	0.45	0.95	0.94	1.34	1.1	**1.30**
Variance	6.93	1.63	2.99	0.2	0.9	0.89	1.79	1.22	**2.07**
18S in VAT									
	Lean L-IR		Lean H-IR		MO L-IR		MO H-IR		MEAN
	Undiff	Diff	Undiff	Diff	Undiff	Diff	Undiff	Diff	
Min	11.23	12.88	12.53	13.39	11.41	10.40	12.96	11.63	12.05
Max	15.65	14.59	15.73	15.85	13.61	16.26	16.31	14.04	15.26
Mean ±SEM	13.15±0.92	13.53±0.37	14.25±0.66	14.39±0.54	12.36±0.46	12.83±1.25	14.45±0.7	12.57±0.52	13.44±0.68
SD	1.84	0.74	1.31	1.07	0.92	2.5	1.39	1.04	**1.35**
Variance	3.39	0.55	1.73	1.16	0.86	6.27	1.94	1.09	**2.12**

## Results

Table 1 shows the clinicopathological data of the study population. As expected, waist circumference and diastolic arterial pressure were statistically increased in morbid obese subjects compared to lean, while HDL cholesterol and adiponectin were decreased. On the other hand, triglycerides levels were statistically increased in lean and morbid obese subjects with high Insulin-Resistance degree (H-IR). In order to determine the expression stability during adipocyte differentiation, human stromal vascular cells (hVSCs) from the ATs were induced to differentiate into adipocytes. hVSCs differentiated into adipocytes, and lipid-filled cells were detected with oil red O-staining (Fig 1A). Moreover, mRNA levels of *PPARγ2* and *FABP4* were increased in differentiated cells from both VAT- and SAT compared to non-differentiated hVSCs (Fig 1B and 1C), and higher levels of *PPARγ2* and *FABP4* mRNA were detected in samples from obese patients when compared to lean subjects, independently of the associated insulin resistance condition (MO L-IR or MO H-IR versus Lean L-IR or Lean H-IR). Additionally, *PPARγ2* levels in VAT were different in each group when compared to any other, with expression levels decreasing in the following way, MO H-IR>MO L-IR>Lean H-IR>Lean L-IR (two-way ANOVA, Bonferroni's post hoc test). The mRNA expression levels of *RPL3A*, *EEF1A1*, *CYC*, *GADPH* and *18S* were assessed in both non-differentiated hVSCs and differentiated adipocytes from the four groups of patients. Analysis of gene expression stability for the candidate reference genes was done by three different approaches, and further validated by BestKeeper analysis and RefFinder platform. First, the raw non-normalized Ct data were analyzed for variability. Tables 2 and 3, show the min, max, mean ± SEM, SD, and variance for each reference gene and type of sample from VAT (Table 2) and SAT (Table 3). *RPL13A* and *EEF1A1* were the less variable genes in VAT with 0.70 and 0.77 SD values and 0.56 and 0.67 variance values, respectively, in contrast to values ranging from 1.25 to 1.35 (SD) and 1.77 to 2.12 (variance) for the other genes. A similar result was found in SAT, with SD values for *RPL13A* and *EEF1A1* of 0.93 and 0.85, respectively (the variance values were 1.08 and 0.87, respectively) in contrast to SD and variance values for the other genes (SD ranged from 1.22 to 1.37 and variance ranged from 1.52 to 2.33). Boxplot representation of these data is shown in Fig 2. Due to the small sample size and statistical purposes, the samples were grouped and compared as lean vs obese, low-IR vs high-IR and VAT vs SAT. Statistical analysis (two-way ANOVA) showed no differences between groups for any of the candidate reference genes.

**Table 3:** Descriptive analysis of Ct values variability from qPCR of undifferentiated and differentiated hVSCs from SAT of patients with different BMI and HOMA-IR index. The table shows the statistics min, max, mean±SEM, standard variation (SD) and variance of the Ct values obtained after qPCR of undifferentiated and differentiated SAT-derived hVSCs in the four groups under investigation as well as the mean values for every reference gene assessed (right columns). Data show that *RPL13A* and *EEF1A1* has the lowest variability (SD and variance values) suggesting more stability in their expression when compared to the other reference genes. VAT: visceral adipose tissue; SAT: subcutaneous adipose tissue. Lean L-IR: lean subjects with low-degree insulin resistance; Lean H-IR: lean subjects with high-degree insulin resistance; MO L-IR: morbid obese subjects with low-degree insulin resistance; MO H-IR: morbid obese with high-degree insulin resistance.

RPL13A in SAT									
	Lean L-IR		Lean H-IR		MO L-IR		MO H-IR		MEAN
	Undiff	Diff	Undiff	Diff	Undiff	Diff	Undiff	Diff	
Min	22.37	23.63	23.06	22.78	22.42	22.35	22.79	22.24	22.70
Max	25.85	24.67	24.06	24.08	24.40	23.69	25.79	25.80	24.79
Mean ±SEM	23.88±0.72	23.96±0.24	23.52±0.23	23.42±0.27	23.49±0.41	23.20±0.31	23.61±0.73	23.80±0.81	23.61±0.46
SD	1.44	0.48	0.45	0.54	0.82	0.62	1.45	1.62	**0.93**
Variance	2.09	0.24	0.21	0.29	0.66	0.39	2.12	2.63	**1.08**
EEF1A1 in SAT									
	Lean L-IR		Lean H-IR		MO L-IR		MO H-IR		MEAN
	Undiff	Diff	Undiff	Diff	Undiff	Diff	Undiff	Diff	
Min	17.28	17.09	17.04	17.54	18.02	17.71	17.30	17.05	17.38
Max	18.31	19.01	18.72	18.78	20.48	21.13	19.65	18.06	19.27
Mean ±SEM	17.87±0.22	17.95±0.44	17.87±0.35	18.19±0.26	18.76±0.58	18.96±0.79	18.10±0.54	17.64±0.21	18.17±0.42
SD	0.43	0.89	0.69	0.52	1.17	1.58	1.08	0.42	**0.85**
Variance	0.19	0.79	0.48	0.27	1.36	2.49	1.18	0.18	**0.87**
CYC in SAT									
	Lean L-IR		Lean H-IR		MO L-IR		MO H-IR		MEAN
	Undiff	Diff	Undiff	Diff	Undiff	Diff	Undiff	Diff	
Min	21.89	23.04	22.79	22.47	22.54	23.06	22.97	23.22	22.75
Max	25.54	26.04	25.14	24.88	25.65	25.27	25.56	25.86	25.49
Mean ±SEM	23.67±0.82	24.38±0.64	23.63±0.54	24.06±0.54	23.82±0.65	24.2±0.56	24.50±0.56	24.56±0.54	24.10±0.6
SD	1.64	1.29	1.09	1.08	1.31	1.12	1.12	1.09	**1.22**
Variance	2.69	1.67	1.18	1.17	1.71	1.26	1.26	1.18	**1.52**
GAPDH in SAT									
	Lean L-IR		Lean H-IR		MO L-IR		MO H-IR		MEAN
	Undiff	Diff	Undiff	Diff	Undiff	Diff	Undiff	Diff	
Min	25.53	22.86	23.96	22.86	25.30	22.88	24.89	24.59	24.11
Max	28.19	26.56	27.08	28.16	25.79	26.24	27.59	27.61	27.15
Mean ±SEM	26.61±0.6	25.06±0.78	25.43±0.64	25.97±1.3	25.60±0.11	24.69±0.72	26.22±0.61	25.98±0.64	25.69±0.67
SD	1.19	1.56	1.29	2.59	0.23	1.44	1.22	1.28	**1.35**
Variance	1.43	2.45	1.67	6.73	0.05	2.07	1.5	1.65	**2.19**
18S in SAT									
	Lean L-IR		Lean H-IR		MO L-IR		MO H-IR		MEAN
	Undiff	Diff	Undiff	Diff	Undiff	Diff	Undiff	Diff	
Min	12.08	10.22	10.34	12.27	11.70	12.40	12.86	12.47	11.79
Max	16.42	13.88	15.45	16.22	14.06	13.54	14.54	13.87	14.75
Mean ±SEM	14.27±1.17	12.47±0.81	13.14±1.14	14.29±0.81	12.83±0.59	13.07±0.26	13.45±0.39	13.16±0.29	13.33±0.68
SD	2.35	1.63	2.28	1.62	1.19	0.52	0.78	0.57	**1.37**
Variance	5.5	2.65	5.22	2.64	1.41	0.27	0.61	0.33	**2.33**

**Fig 3:**
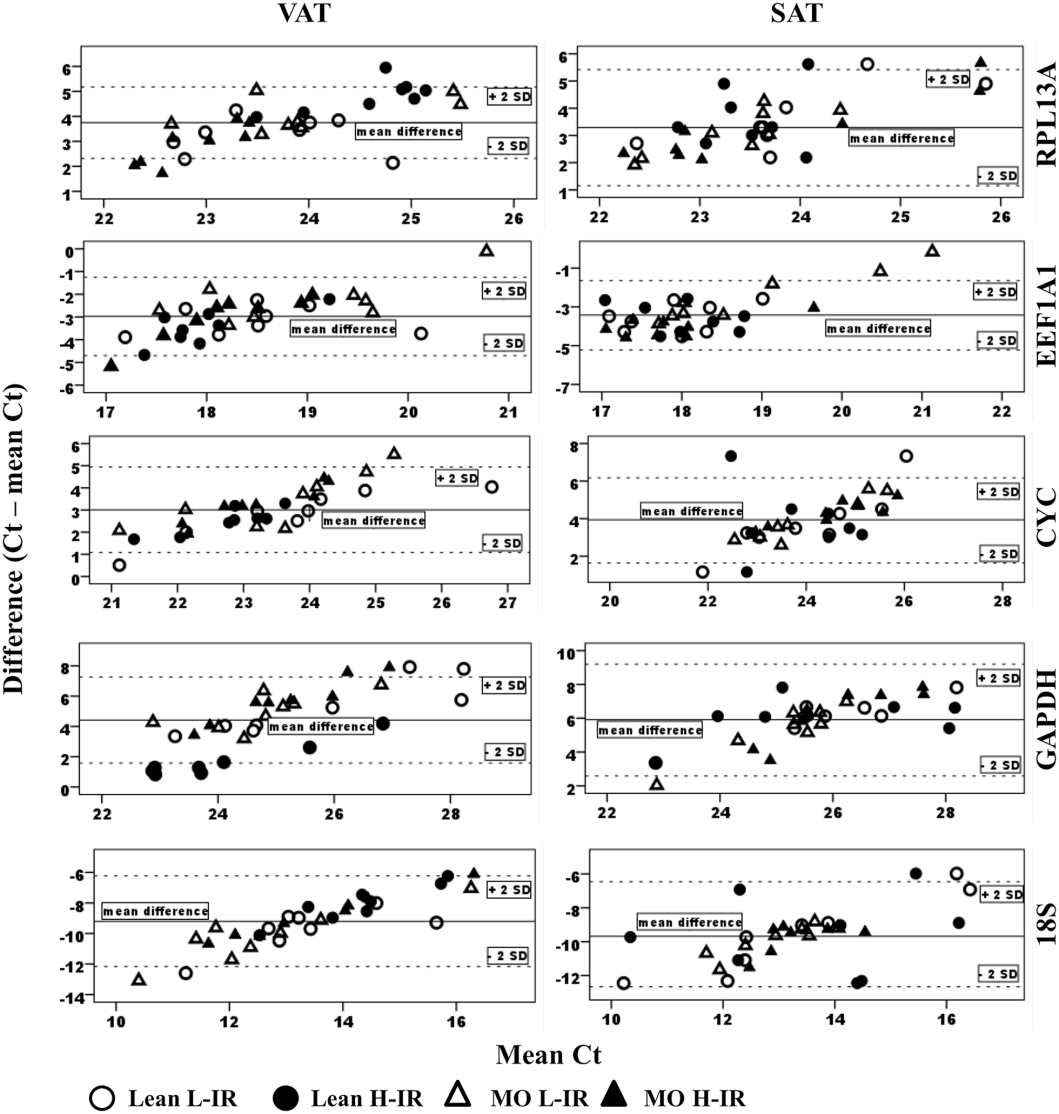
Variability of gene expression among groups and fat depots. Raw non-normalized Ct values from qPCR were collected and processed in the following way: Difference of Ct from *RPL13A*, *EEF1A1*, *CYC*, *GAPDH* and *18S* to the average of the remaining genes was calculated for all samples from lean L-IR (white circles), Lean H-IR (black circles), MO L-IR (white triangles) and MO H-IR (black triangles) in VAT and SAT and plotted for a visual comparison of samples among groups. Full line represents mean difference and dashed lines illustrate precision as 2-fold standard deviation (±2SD). VAT: visceral adipose tissue; SAT: subcutaneous adipose tissue. Lean L-IR: lean subjects with low-degree insulin resistance; Lean H-IR: lean subjects with high-degree insulin resistance; MO L-IR: morbid obese subjects with low-degree insulin resistance; MO H-IR: morbid obese with high-degree insulin resistance. For further information see also Table 4.

Subsequently, to compare the expression levels of each reference gene, their mean difference value (MD) were calculated as the difference of the Ct with the mean expression of the other genes (mean Ct), and illustrated by plotting of the individual MD-value against mean Ct (Fig 3). Precision is illustrated by the 2-fold standard deviation (±2SD). Table 4 shows in more detail the level of expression for each reference gene among the different groups and adipose depots (MD values and ±2SD). Statistical analysis (one-way ANOVA) showed no differences between groups for any gene. Regarding the expression level, in VAT and SAT, the 18S showed the highest expression, with an MD-value of -9.17 and -9.56 (for VAT and SAT, respectively) and *GAPDH* had the lowest expression level, with a MD-value of +5.22 and +5.89 (VAT and SAT, respectively). Expression levels in VAT were 18S>*EEF1A1*>*CYC*> *RPL13A*>*GAPDH*, and in SAT were 18S>*EEF1A1*>*RPL13A*>*CYC*>*GAPDH*. Precision ranged from 1.39 for *RPL13A* to 2.86 for *18S* in VAT and from 1.67 for *EEF1A1* to 2.85 for *GAPDH* in SAT. Thus, despite being highly expressed, *18S* had the lowest precision in VAT and also a low precision in SAT (2.77). By contrast, *EEF1A1* had a high expression in VAT and SAT in combination with a high precision (low±2SD values). *RPL13A*, despite having moderate expression in VAT and SAT also showed a high precision in both VAT and SAT (1.39 and 1.79 for ±2SD values, respectively).

**Table 4:** Mean difference values and precision of gene expression in samples from patients with different BMI and HOMA-IR index. The mean difference (MD) for each sample was calculated as the difference of Ct from *RPL13A*, *EEF1A1*, *CYC*, *GAPDH* and *18S* to the average of the other four genes and tissues. Then, the difference was averaged separately for each group (Lean L-IR, Lean H-IR, MO L-IR, MO H-IR). The right column represents the mean values for the four groups. The precision was calculated as 2-fold standard deviation (±2SD). No statistical differences for MD were found among groups (one-way ANOVA). Expression levels for VAT were *18S>EEF1A1>CYC>RPL13A>GAPDH*, and those of SAT were *18S>EEF1A1>RPL13A>CYC>GAPDH*. Precision ranged from 1.39 for *RPL13A* to 2.86 for *18S* in VAT and from 1.67 for *EEF1A1* to 2.85 for *GAPDH* in SAT. VAT: visceral adipose tissue; SAT: subcutaneous adipose tissue. Lean L-IR: lean subjects with low-degree insulin resistance; Lean H-IR: lean subjects with high-degree insulin resistance; MO L-IR: morbid obese subjects with low-degree insulin resistance; MO H-IR: morbid obese with high-degree insulin resistance.

	VAT									
Gene	Lean L-IR		Lean H-IR		MO L-IR		MO H-IR		MEAN	
	MD	± 2 SD	MD	± 2 SD	MD	± 2 SD	MD	± 2 SD	MD	± 2 SD
RPL13A	3.26	1.55	4.82	1.16	4.06	1.93	2.86	0.92	3.75	1.39
EEF1A1	-3.14	1.74	-3.47	1.12	-2.26	2.15	-3.03	1.35	-2.98	1.59
CYC	3.45	3.41	2.52	1.49	3.44	2.75	3.28	1.79	3.17	2.36
GAPDH	6.00	3.85	4.16	2.86	5.00	2.25	5.73	2.33	4.42	2.82
18S	-9.56	2.63	-8.03	2.23	10.23	3.53	-8.84	3.03	-9.17	2.86
	SAT									
Gene	Lean L-IR		Lean H-IR		MO L-IR		MO H-IR		MEAN	
	MD	± 2 SD	MD	± 2 SD	MD	± 2 SD	MD	± 2 SD	MD	± 2 SD
RPL13A	3.63	2.00	3.15	0.93	3.10	1.38	3.26	2.86	3.29	1.79
EEF1A1	-3.88	1.29	-3.65	1.19	-2.50	2.58	-4.04	1.61	-3.51	1.67
CYC	3.77	2.84	3.62	2.06	3.94	2.29	4.28	2.05	3.90	2.31
GAPDH	6.03	3.07	5.94	3.84	5.35	2.14	6.25	2.34	5.89	2.85
18S	-9.55	4.20	-9.07	3.85	-9.89	1.72	-9.75	1.31	-9.56	2.77

**Fig 4:**
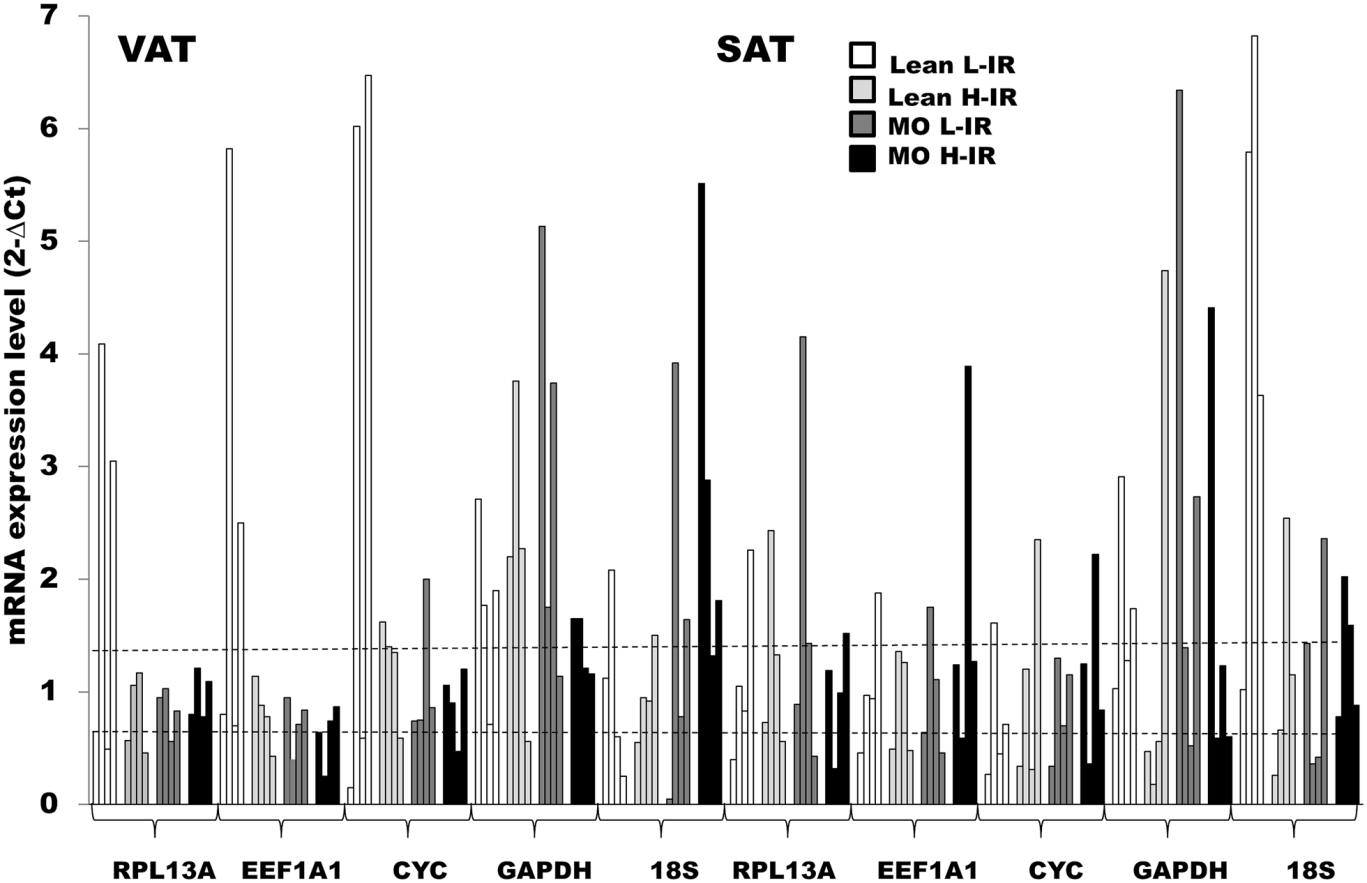
Gene expression stability during differentiation in VAT and SAT samples from subjects with different BMI and HOMA-IR index. hVSCs from VAT and SAT of subjects with different BMI and HOMA-IR index were induced to differentiate, and mRNA expression of *RPL13A*, *EEF1A1*, *CYC*, *GAPDH* and *18S* was analyzed by qPCR. Change in gene expression during differentiation was estimated by calculating 2^-ΔCt^, where ΔCt = (mean Ct in differentiated sample—mean Ct in undifferentiated sample). Dashed lines represent limits for 2^-ΔCt^ acceptable values; those that fall in between 0.7 and 1.4 can be considered fluctuation in gene expression that are due to technical variance. Each bar represents mean expression in one single sample. Error bars have been omitted for clarity purposes. It can be seen that in both VAT and SAT the number of samples within the acceptable range is higher for *RPL13A*, *EEF1A1* and *CYC* than for *GAPDH* and *18S*. VAT: visceral adipose tissue; SAT: subcutaneous adipose tissue. Lean L-IR: lean subjects with low-degree insulin resistance; Lean H-IR: lean subjects with high-degree insulin resistance; MO L-IR: morbid obese subjects with low-degree insulin resistance; MO H-IR: morbid obese with high-degree insulin resistance.

We assessed variability of gene expression during differentiation by comparing the ΔCt values in samples from the same patients before and after differentiation (Fig 4). The criterion of ΔCt value ≤ ± 0.5 was used as a delimiter of reference gene suitability during differentiation. All data were converted into fold-changes using the formula 2^-ΔCt^; and, accordingly, values that filled in between 0.7 and 1.4 suggested that there were no significant differences between undifferentiated and differentiated adipocytes (Fig 4). Among the five reference genes, *RPL13A*, *EEF1A1* and *CYC* were the most stable reference genes during differentiation in both VAT and SAT, as most samples displayed fold-changes values between 0.7 and 1.4. In fact, *RPL13A* and *EEF1A1* were the most stable with 10 and 11 out of 16 samples (for VAT) and 8 and 9 out of 16 samples (for SAT) within this range. By contrast, *GAPDH* and 18S were the less stable, showing only 4 and 6 out of 16 samples (for VAT and SAT) within this range.

Validation of these findings was done by BestKeeper analysis and by using the RefFinder platform. Tables 5 and 6 show the descriptive and regression analysis, respectively, after the BestKeeper processing. Table 5 shows that, when analyzing the five reference genes in a separate manner (left column), *GAPDH* and *18S* have a SD>1. Moreover, they have a [X-fold] parameter in the regression analysis >2 (Table 6), indicating that both are unsuitable reference genes. Correlation analysis showed a wide range in the Pearson correlation coefficient (0.674<*r* <0.937), suggesting that *GAPDH* and *18S* should not be considered as suitable (Table 6). Subsequently, when *CYC*, *RPL13A* and *EEF1A1* were analyzed together by BestKeeper; the SD was < to 1 (Table 5, middle column), the Pearson correlation range fill in between 0.855 and 0.931 (Table 6) and *CYC* showed X-fold parameter >2 (Table 6), suggesting that *CYC* should also be removed.

**Table 5:** Descriptive statistics of the five candidate reference genes, calculated BestKeeper Index and comparative analysis with RefFinder. Data of expression for the five candidate reference genes (RGs), based on their crossing point (CP) values, were introduced in BestKeeper software. The result of descriptive analysis for every gene is showed in the left column. The Bestkeeper index was calculated for five genes (*RPL13A*, *EEF1A1*, *CYC*, *GAPDH* and *18S*), for three genes (*RPL13A*, *EEF1A1* and *CYC*) and for two genes (*RPL13A* and *EEF1A1*) (middle column). Data for the combination of 5 and 3 RGs were introduced in the RefFinder tool and the results are shown in the right column. Geometric Mean (GM), Arithmetic Mean (AM), Minimum (Min) and Maximum (Max) Crossing Point value (CP), Standard Deviation (SD) and Coefficient of Variation (CV) * indicates when the value is out of range.

	BESTKEEPER: DESCRIPTIVE ANALYSIS (CP)					BESTKEEPER INDEX			RANKING REFFINDER		
	RPL13A	EEF1A1	CYC	GAPDH	18s	5 RG	3 RG	2 RG	RG	5 RG	3 RG
**GM**	**23.64**	**18.26**	**23.5**	**25.3**	**12.9**	**20.7**	**21.81**	**20.77**	**RPL13A**	**1.86**	**1.41**
**AM**	**23.65**	**18.27**	**23.6**	**25.34**	**13.16**	**20.8**	**21.83**	**20.79**	**EEF1A1**	**1.68**	**1.19**
**Min**	**22.24**	**17.05**	**21.1**	**22.86**	**4.4**	**17.5**	**20.14**	**19.47**	**CYC**	**1.73**	**3**
**Max**	**26.02**	**21.13**	**26.8**	**28.19**	**16.42**	**23.7**	**24.64**	**23.45**	**GAPDH**	**3.72**	
**SD ±**	**0.49**	**0.48**	**0.85**	1.07 *	1.31*	**0.84**	**0.61**	**0.48**	**18s**	**5**	
**CV %**	**2.06**	**2.61**	**3.61**	**4.24**	**9.98**	**4.5**	**2.77**	**2.3**			

**Table 6:** Regression Analysis from the BestKeeper. Data represent pair-wise correlation analysis of the reference genes (RGs) versus the BestKeeper index (BK) of 5.3 or 2 RGs. The first five lines represent the analysis of every gene versus the BK calculated for the five genes. Next three lines represent the analysis of every gene versus the BK calculated for the three genes. Last two lines represent the analysis of every gene versus the BK calculated for the two genes.

RG vs. BK	PEARSON'S COEFF. [r]	COEFFICIENT of DETERM. [r^2^]	INTERCEPTION [CP]	SLOPE [CP]	SE [CP]	p-value	POWER [X-fold]
**RPL13A vs. BK (5RG)**	**0.674**	**0.454**	**12.237**	**0.561**	**± 0.545**	**0.001**	**1.47**
**EEF1A1 vs. BK (5RG)**	**0.715**	**0.511**	**5.702**	**0.617**	**± 0.536**	**0.001**	**1.53**
**CYC vs. BK (5RG)**	**0.937**	**0.878**	**-1.259**	**1.223**	**± 0.404**	**0.001**	2.33*
**GAPDH vs. BK (5RG)**	**0.696**	**0.484**	**3.823**	**1.063**	**± 0.973**	**0.001**	2.09*
**18S vs. BK (5RG)**	**0.833**	**0.694**	**-12.666**	**1.286**	**± 0.757**	**0.001**	2.44*
**RPL13A vs. BK (3RG)**	**0.92**	**0.846**	**5.186**	**0.852**	**± 0.286**	**0.001**	**1.81**
**EEF1A1 vs. BK (3RG)**	**0.931**	**0.867**	**-1.16**	**0.897**	**± 0.277**	**0.001**	**1.86**
**CYC vs. BK (3RG)**	**0.855**	**0.731**	**-3.646**	**1.256**	**± 0.599**	**0.001**	2.39*
**RPL13A vs. BK (2RG)**	**0.989**	**0.978**	**3.529**	**0.968**	**± 0.108**	**0.001**	**1.96**
**EEF1A1 vs. BK (2RG)**	**0.994**	**0.988**	**-2.771**	**1.012**	**± 0.083**	**0.001**	**2.02**

* indicates that the gene showed an [x-fold] out of range (>2). *GAPDH* and *18S* were discarded after the first analysis. *CYC* was discarded after the second analysis.

Next, when *RPL13A* and *EEF1A1* were analyzed together; the SD was <1 (Table 5, middle column) and the X-fold was 2 (Table 6). Repeated pair-wise correlation analysis showed a high correlation of these reference genes vs BestKeeper, showing a narrow range of 0.989<*r*<0.994. Taken together, these data indicate that *RPL13A* and *EEF1A1* are good reference genes and they could be used alone or in combination for the analysis of gene expression changes in human VAT and SAT during adipogenesis.

In order to further validate our results from the BestKeeper analysis, we used the RefFinder platform, which is a popular tool for reference gene validation that performs a quick analysis using the four most popular algorithms for reference gene validation (GeNorm, BestKeeper, NormFinder and ΔCt method), starting from a single input of the Ct values. In this case, we obtained similar results than that of the BestKeeper (Table 5, right column). The first analysis of the five reference genes led to a similar ranking for *RPL13A*, *EEF1A1* and *CYC*, suggesting that *GAPDH* and *18S* should be removed. Subsequent analysis of the three selected reference genes led to removal of *CYC*, indicating that *RPL13A* and *EEF1A1* were the most suitable genes and thus confirming our previous results.

## Discussion

qPCR gene expression analysis is a widely used technique to determine differences in gene expression between samples [26]. To exclude any artifactual interpretation, the technique requires solid normalization strategies. Among the available normalization methods, the use of reference genes is currently the preferred method [27]. The use of inappropriate reference genes is a widely accepted cause of misinterpretation of the results [28]. The expression stability of the reference gene determines the sensitivity and reliability of mRNA quantification by qPCR [29]. Notably, studies of well-known reference genes such as *GADPH* and *β*-*ACT* show considerable variation in their expression levels depending on the tissue type and experimental conditions [30]. Particularly, in VAT and SAT, the expression of these genes is unstable while comparing samples from healthy subjects with those from patients with obesity and type 2 diabetes mellitus [31], this thus highlighting the need of validated reference genes for these sort of studies. Also, a study in human epicardial AT from lean, overweight and obese subjects identified *CYCA*, *GAPDH* and *RPL27* as the most stable genes [32]. To the best of our knowledge, there are current no validated reference genes for studies performed in human hVSCs from VAT and SAT isolated from subjects with different BMI and/or HOMA-IR. Here, we analyzed for the first time five putative reference genes (*RPL13A*, *EEF1A1*, *CYC*, *GAPDH* and *18S*), commonly used for the analysis of adipocyte differentiation from hVSCs or cell lines. hVSCs from VAT and SAT isolated from subjects with different BMI and/or HOMA-IR were differentiated into adipocytes. Both oil red O-staining and *FABP4/PPARγ2* mRNA expression analyzes confirmed the successful adipocyte differentiation. On the other hand, even if *PPARγ2* and *FABP4* mRNA expression levels were found to be higher in samples from obese patients (two-way ANOVA) compared to those from leans, it still debatable to confirm or to rule out that hVSC from obese subjects displayed higher adipocyte differentiation compared to lean, due to the small sample number used in this study. Further analysis with greater sample number should be carried out to investigate this fact. It is relevant to highlight that the goal of this study was to analyze whether the level of adipocyte differentiation might affect the variance of the reference genes. Thus, suitable reference genes should be those not affected by changes in the level of differentiation.

We have performed our own analysis of gene expression level, variability among groups and gene expression stability by analyzing raw non-normalized Ct values and change in Ct values during differentiation. Furthermore, we have validated our findings by using BestKeeper analysis and the RefFinder platform. Taken together, the analysis suggests that *RPL13A* and *EEF1A* are the most reliable and stable reference genes for these tissues and patient types. The Bestkeeper analysis also suggested that the combination of these two reference genes can provide a more robust method than using them separately.

Our findings agree with a previous study showing that *RPL13A* is the most stable reference gene for adipose tissue- and Wharton’s Jelly-derived human MSCs expansion and differentiation analysis [11]. In fact, *RPL13A* in combination with *GAPDH* [13] or *EEF1A1* and *18S* [15] has been found to be appropriate for the analysis of gene expression during adipogenesis of mesenchymal stem cells from bone marrow cells [13,15] or VAT 11]. On the other hand, the 18S has been used as reference gene for SAT [16,17]. However, there are no studies validating it. Our study shows that changes in the expression of the 18S gene during adipocyte differentiation are higher than expected, thus suggesting that it is not suitable as reference gene in this process. By contrast, our study confirms that *RPL13A* and *EEF1A1* are stable reference genes for VAT or SAT samples from subjects with different degrees of obesity and IR. Furthermore, we found that, together with *18S*, *GAPDH* and *CYC* were not suitable reference genes for these experimental conditions, which is in agreement with other study [33]. Another study also found that the combination of *18S*, *EF1-α* and *β-ACT* is not suitable for the analysis of VEGF-treated human MSCs from bone marrow and AT [34]. Our data also support the combined use of reference genes, which is in agreement with a previous report [35].

We could not detect significant changes in the expression level among groups for any of the reference genes studied. However, we cannot rule out this possibility, because the sample size is small when the patients are classified according to body weight and HOMA-IR index (n = 4 each group). However, the analysis of Ct values for lean vs obese (n = 8), low-IR vs high-IR (n = 8) and VAT vs SAT (n = 16) did not either detect changes between them.

In conclusion, we show for the first time that *EEF1A1* and *RPL13A* are stable reference genes during adipogenic differentiation of VAT and SAT human samples. Thus, they are suitable for gene expression studies of VAT and SAT-derived stem cells from subjects with different degrees of obesity and IR. The use of these genes in pair combinations may further enhance the strength of the data obtained from gene expression analysis in this cellular system.

## Supporting Information

S1 Table TaqMan^®^ probe’s references from Applied Biosystems. (DOCX)

## Abbreviations

qPCR: quantitative Polymerase Chain Reaction
hVSCs: human Vascular Stromal Cells
BMI: Body Mass Index
HOMA-IR: Homeostatic Model Assessment of Insulin Resistance index
RG: Reference Gene
TG: Target Gene
BK: BestKeeper index
GAPDH: glyceraldehyde-3 phosphate dehydrogenase
CYC: cyclophilin A
RPL13A: Ribosomal Protein L13A
EEF1A1: eukaryotic translation elongation factor 1 alpha 1
18S: eukaryotic 18S ribosomal rRNA
