# Supplementary material for: RPL13A and EEF1A1 Are Suitable Reference Genes for qPCR during Adipocyte Differentiation of Vascular Stromal Cells from Patients with Different BMI and HOMA-IR
Source: PLoS One. 2016 Jun 15;11(6):e0157002. doi: 10.1371/journal.pone.0157002 (PMC4909211; doi:10.1371/journal.pone.0157002)
Supplement: S1 Table — (DOCX) [file pone.0157002.s001.docx]

**SUPPLEMENTARY DATA**

**S1 Table. TaqMan^®^ probe’s references from Applied Biosystems**

| **GENE** | **REFERENCE** | **Sequence identity (NCBI database)** | **AMPLICON** |
| --- | --- | --- | --- |
| **Cyclophilin A (CYC)** | **4326316E** | **NM_021130.3** | **98** |
| **Glyceraldehyde-3 phosphate dehydrogenase (GAPDH)** | **Hs99999905_m1** | **NM_002046.3** | **122** |
| **Ribosomal protein L13A (RPL13A)** | **Hs04194366** | **NM_001270491.1** | **113** |
| **Eukaryotic translation elongation factor 1 alpha 1 (EEF1A1)** | **Hs00265885_g1** | **NM_001402.5** | **75** |
| **Eukaryotic 18s rRNA (18S)** | **Hs99999901_s1** | **NM_03205.1** | **187** |
| **Peroxisome proliferator-activated receptor gamma (PPARɤ)** | **Hs01115510_m1** | **NM_015869.4** | **92** |
| **Faty acid binding protein 4, adipocyte (FABP4)** | **Hs01086177_m1** | **NM_001442.2** | **96** |
